# Smartphone App Delivery of a Just-In-Time Adaptive Intervention for Adult Gamblers (Gambling Habit Hacker): Protocol for a Microrandomized Trial

**DOI:** 10.2196/38919

**Published:** 2022-07-26

**Authors:** Simone N Rodda, Kathleen L Bagot, Stephanie S Merkouris, George Youssef, Dan I Lubman, Anna C Thomas, Nicki A Dowling

**Affiliations:** 1 Psychology and Neuroscience Auckland University of Technology Auckland New Zealand; 2 School of Psychology Deakin University Geelong Australia; 3 School of Population Health University of Auckland Grafton New Zealand; 4 Data Drawer Consulting Sandringham Australia; 5 Turning Point and Monash Addiction Research Centre, Eastern Health Clinical School, Monash University Melbourne Australia; 6 Melbourne Graduate School of Education University of Melbourne Parkville Australia

**Keywords:** Just-In-Time Adaptive Intervention, JITAI, ecological momentary assessment, EMA, ecological momentary intervention, EMI, gambling, behavior change technique, implementation intentions, action planning, coping planning, microrandomized trial, mobile phone

## Abstract

**Background:**

People with gambling problems frequently report repeated unsuccessful attempts to change their behavior. Although many behavior change techniques are available to individuals to reduce gambling harm, they can be challenging to implement or maintain. The provision of implementation support tailored for immediate, real-time, individualized circumstances may improve attempts at behavior change.

**Objective:**

We aimed to develop and evaluate a Just-In-Time Adaptive Intervention (JITAI) for individuals who require support to adhere to their gambling limits. JITAI development is based on the principles of the Health Action Process Approach with delivery, in alignment with the principles of self-determination theory. The primary objective was to determine the effect of action- and coping planning compared with no intervention on the goal of subsequently adhering to gambling expenditure limits.

**Methods:**

*Gambling Habit Hacker* is delivered as a JITAI providing in-the-moment support for adhering to gambling expenditure limits (primary proximal outcome). Delivered via a smartphone app, this JITAI delivers tailored behavior change techniques related to goal setting, action planning, coping planning, and self-monitoring. The *Gambling Habit Hacker* app will be evaluated using a 28-day microrandomized trial. Up to 200 individuals seeking support for their own gambling from Australia and New Zealand will set a gambling expenditure limit (ie, goal). They will then be asked to complete 3 time-based ecological momentary assessments (EMAs) per day over a 28-day period. EMAs will assess real-time adherence to gambling limits, strength of intention to adhere to goals, goal self-efficacy, urge self-efficacy, and being in high-risk situations. On the basis of the responses to each EMA, participants will be randomized to the control (a set of 25 self-enactable strategies containing names only and no implementation information) or intervention (self-enactable strategy implementation information with facilitated action- and coping planning) conditions. This microrandomized trial will be supplemented with a 6-month within-group follow-up that explores the long-term impact of the app on gambling expenditure (primary distal outcome) and a range of secondary outcomes, as well as an evaluation of the acceptability of the JITAI via postintervention surveys, app use and engagement indices, and semistructured interviews. This trial has been approved by the Deakin University Human Research Ethics Committee (2020-304).

**Results:**

The intervention has been subject to expert user testing, with high acceptability scores. The results will inform a more nuanced version of the *Gambling Habit Hacker* app for wider use.

**Conclusions:**

*Gambling Habit Hacker* is part of a suite of interventions for addictive behaviors that deliver implementation support grounded in lived experience. This study may inform the usefulness of delivering implementation intentions in real time and in real-world settings. It potentially offers people with gambling problems new support to set their gambling intentions and adhere to their limits.

**Trial Registration:**

Australian New Zealand Clinical Trials Registry ACTRN12622000497707; www.anzctr.org.au/Trial/Registration/TrialReview.aspx?id=383568

**International Registered Report Identifier (IRRID):**

DERR1-10.2196/38919

## Introduction

### Background

Gambling disorder is classified as an addiction and related disorder in The Diagnostic and Statistical Manual of Mental Disorders, Fifth Edition. It is characterized by repeated unsuccessful attempts to change behavior, loss of control, and the development of tolerance and withdrawal symptoms [1]. Problem gambling is a commonly used term in many jurisdictions, such as Australia and New Zealand, to denote gambling that negatively impacts the gambler as well as their family, friends, and the community [2]. Worldwide, prevalence estimates of past-year problem gambling in adults have ranged from 0.1% to 5.8% over the last decade [3]. In Australia and New Zealand, approximately 0.4% to 0.7% of adults report past-year problem gambling, with an additional 2% to 11% reporting moderate-risk gambling and 3% to 7.7% reporting moderate-risk gambling [4-6]. Although a relatively low prevalence disorder, recent estimates have indicated that the burden of harm associated with gambling problems in the population is relatively high [7] and can include a range of financial, relationship, and psychological harms [8]. Moreover, although people with problem gambling experience more individual harm than people at lower risk for problem gambling, it has been estimated that 85% of the total burden of harm can be attributed to people with low- and moderate-risk gambling because of their greater prevalence in the population [7]. Gambling problems are also highly comorbid with other addictive behaviors such as nicotine and alcohol use and mental health disorders, including anxiety, depression, and personality disorders [9,10]. Global estimates of help seeking indicate 1 in 25 moderate-risk gamblers and 1 in 5 people with problem gambling have sought help for problems related to their gambling [11].

There is evidence that problem gambling can be responsive to treatment, with the most efficacious interventions being cognitive behavioral therapy and, to a lesser extent, motivational interviewing [12,13]. These interventions include a range of professionally derived behavior change techniques (BCTs) [14]. These techniques are the theorized active ingredients of behavior change interventions that can be observed and replicated [14]. Research examining the components of gambling interventions identified 18 categories of techniques, including cognitive restructuring, behavior substitution, stimulus control, social support, and self-monitoring [15]. In addition to these professionally derived techniques, studies indicate that people with gambling problems select and implement similar techniques without professional oversight [15-20]. Many gamblers attempt to reduce their gambling behavior by setting expenditure, frequency, and time limits [21]. Research on gamblers has identified 15 different categories of self-enacted strategies used to adhere to gambling limits [22]. Gamblers also use strategies before gambling (eg, setting a limit), while gambling (eg, placing low-value bets), and after gambling (eg, having a plan on when to walk away) [20,23,24].

Gamblers may implement strategies to limit or reduce gambling behaviors, but high rates of relapse suggest that these are not always successful in the long term [25]. Variable success may be due to a failure to select a specific strategy fit for a purpose, shifting priorities, an inability to maintain the approach, or implementing conflicting strategies [26]. Advice to individuals on how to adhere to gambling limits is available, but this is limited to brief information such as “Set a money limit in advance” and “Exercise control over your gambling” [20]. Gamblers find it challenging to adhere to limits, and knowing how to implement these strategies may be difficult. For example, in-venue messaging may suggest taking a break, but details as to when, where, and for how long to take a break are not broken down or personalized. Messages such as *gamble responsibly* may be too broad to be easily applied, especially when an individual is already in a venue gambling or when gambling urges are intense. When these strategies have previously been delivered as part of an intervention, gamblers have recommended individual tailoring by matching strategy and motivation or situation [27]. These findings are consistent with self-determination theory, which posits that behavior change occurs when an individual is intrinsically motivated and able to drive their own change through self-selection and enactment of self-management strategies, as well as the enhancement of competence and self-efficacy [28,29]. The value of interventions specifically designed to support gamblers in implementing strategies to reduce gambling-related harm has also been previously identified [23,30].

### Planning Techniques for Gambling Reduction

People with gambling problems experience repeated failed attempts to change their behavior [1]. Research indicates that this may be owing to implementation failure, whereby good intentions have not consistently led to intended actions [24,26]. Social cognitive theories such as the theory of planned behavior focus on factors that predict intention, including attitudes, perceived behavioral control, and subjective norms, on the basis that intention predicts subsequent behavior [31]. Although meta-analyses indicate a strong relationship between intention and behavior, accounting for more than one-fourth (27%) of the variance in health behavior change [31], there appears to be a gap between intention to perform a behavior and successful implementation of that behavior. To address this gap, researchers have developed social cognitive models such as the Health Action Process Approach (HAPA) [32]. HAPA proposes that behavior change follows a continuous 2-phase process that involves motivation and volition. In this model, motivation refers to forming an intention by realizing that a particular behavior needs to change, that such change would be worthwhile and should be prioritized over and above other competing demands, and that the individual can implement the selected action (task self-efficacy) [33]. The volitional phase facilitates forward movement toward implementing intentions with techniques such as action planning, coping planning, and self-monitoring [34-36]. Factors that can help or hinder the implementation of intentions in the volitional phase include maintenance self-efficacy (belief in the ability to maintain plans and cope with barriers that arise) and recovery self-efficacy (belief in the ability to regain control after failure to cope with implementation barriers) [36].

Overall, 2 implementation planning techniques for addressing this gap have been subject to extensive evaluation across a range of health behaviors: action planning and coping planning. Action- and coping planning are BCTs [14] that can be delivered independently or combined with other BCTs, such as rewards, social support, or environmental restructuring, to form a multicomponent intervention. Action planning outlines how, when, and where a specific behavior will be implemented [36], whereas coping planning pre-empts barriers to implementing the desired behavior, developing an if-then plan. The if-then plan links specific situations or events with a detailed plan that can be implemented when a situation or barrier to implementing the behavior is present [34]. Action- and coping planning require little effort and can be easily personalized for each individual.

Recent meta-analyses have shown that action- or coping planning successfully improves addiction-related behaviors such as smoking and alcohol use [37,38]. For example, a review of 12 randomized controlled trials revealed that the use of action- or coping planning (pen-and-paper or web-based delivery) significantly improved smoking cessation rates [37]. Overall, the attrition rates were high, and there were few follow-up periods beyond 2 months. Another meta-analytic review, including 15 randomized controlled trials, reported that planning displayed a small to medium effect size in reducing alcohol use after treatment compared with active and passive control conditions [38]. Although the results from studies with low methodological quality were retained in the analysis, these findings support the need to establish the effectiveness of these interventions.

Preliminary work has examined the use of action- and coping planning by gamblers to support the successful implementation of goals in a real-world setting [39]. A brief intervention by Rodda et al [39] comprised individually set expenditure goals (intended expenditure set before gambling); tailored action plans that detail how, when, and where strategy is implemented; and coping plans that detail what to do if a barrier to implementing a strategy occurs. This gambling venue-based study reported substantial reductions in actual venue expenditure compared with intended expenditure for people with moderate risk and problem gambling but not for people with nonproblem or low-risk gambling. Notably, 50% of the total sample (intervention and assessment-only control) reported a plan to be implemented before coming to the venue. However, more than two-thirds (69%) of the intervention group were unable to complete a coping plan despite being prompted by the researcher. During the development of plans, gamblers indicated that they could not envisage specific barriers to implementation. Planning techniques, by design, are intended to be completed in advance to link internal states such as urge and situational cues such as being near a venue with a prespecified and semiautomatic action [36,40]. Planning for internal and situational cues is problematic when barriers cannot be identified.

Taken together, these findings indicate that addictive behaviors may be difficult to change owing to their complexity and multiple internal or situational cues for the behavior. As such, single plans may be insufficient because they cannot cover an array of relevant or unidentified cues that can affect motivation and volition [41]. However, having multiple action or coping plans may not effectively address behavior, because the advanced development of action plans for all possible internal or situational cues is not feasible. Furthermore, the likelihood of multiple plans being effective is reduced because of the cognitive burden of retaining and activating the details of multiple plans [41,42]. Just-In-Time Adaptive Intervention (JITAI) approaches may be effective for delivering the range of plans needed to address varying internal or situational cues that render self-regulation challenges.

### JITAI Approach

JITAIs, which use computer algorithms to decide when and how support is provided, address dynamically changing individual needs by providing the type and amount of support required at the right time and only when needed [43]. Nahum-Shani et al [43] describe several key components to guide the design of JITAIs: *decision points*, which refer to the points in time at which decisions about intervention delivery are made; *intervention options*, which include the type, timing, dose, and delivery mode of support that can be delivered at each decision point; *tailoring variables*, which are defined as those that collect internal state or ecological context to decide when or how interventions are delivered; and *decision rules*, which determine which intervention options to offer, for whom, and when at different levels of each tailoring variable. Ecological momentary assessments (EMAs) [44] can be used to provide a real-time evaluation of a person’s current internal and situational cues through mini-assessments delivered via smartphones multiple times per day. The JITAI design is guided by a distal outcome, which is a long-term goal achieved via changes to proximal outcomes, which are short-term goals [43].

JITAIs are effective for a range of health and mental health outcomes, with a recent meta-analysis reporting moderate to large effects for improvements in a range of outcomes, including mental health, diet or weight loss, and physical activity, when compared with wait-list controls and non-JITAI treatments [45]. JITAIs for the treatment and recovery of addictions such as tobacco, alcohol, and drug use show promise [46], while others are underway [47]. In the gambling field, 2 smartphone JITAIs have proposed using geolocation sensors to notify gamblers of situational cues [48,49]. One of these apps, which notified users when in proximity to gambling venues, has been partially evaluated. Humphrey et al [48] conducted a focus group of potential users who reported an interest in the app but low uptake or retention due to high battery use. In addition, 2 smartphone apps that use EMA to identify internal or situational cues for gambling have been developed [50-52]. Compared with geolocation sensors, smartphone JITAIs using EMAs are unlikely to have an impact on battery use. However, of these smartphone JITAIs, only one has been evaluated, with Merkouris et al [50] developing and evaluating a JITAI targeting gambling cravings and reporting high ratings for app helpfulness, usability, and improvements in time-related craving intensity [52]. They also reported medium to large effects for improved gambling symptom severity, cravings, frequency, and gambling expenditure. To date, JITAIs have delivered planning interventions for alcohol reduction [53,54], but no JITAI has delivered tailored action- and coping planning interventions for gambling, despite research testing planning interventions for gamblers recommending the use of in-the-moment support in delivering such interventions [39].

### Research Aims and Hypotheses

This protocol presents the development and evaluation of a theoretically derived JITAI for people who want support in adhering to their gambling expenditure limits. *Gambling Habit Hacker* is a smartphone-delivered JITAI informed by HAPA and the implementation intention literature and delivered in accordance with self-determination theory. This JITAI uses decision rules specifying that participants who are receptive to treatment and report low strength of goal intention, low goal self-efficacy, low urge self-efficacy, or a high-risk situation (tailoring variables) in time-based EMAs sent during 3 semirandom times a day are delivered action- and coping planning activities to implement selected behavior change strategies. Implementation support includes providing a tailored set of self-enactable cognitive and behavioral strategies derived from data synthesis of lived experience [17,22,23,26]. These components are guided primarily by the long-term goal of reducing gambling expenditure (distal outcome), which is posited to be achieved through the short-term goal of adhering to gambling expenditure limits (primary proximal outcome) through increased strength of intention, goal self-efficacy, and urge self-efficacy (secondary proximal outcomes).

This protocol describes the theoretical basis of the intervention and research design of a 28-day microrandomized trial (MRT). An MRT design is a form of sequential factorial design in which each individual is randomized to intervention options at each decision point across a period of weeks or months [55]. In this MRT, each participant will be randomized to an action- and coping planning intervention that responds immediately to real-time implementation barriers (and therefore helps individuals adhere to gambling expenditure limits) and a control condition involving the presentation of a set of 25 self-enactable strategy groups alone (strategy group names only without any implementation guidance). The results will inform the optimization of future versions of the intervention [55].

The primary aim of the 28-day MRT was to determine the efficacy of action- and coping planning versus control on goal adherence. Goal adherence is the primary proximal outcome and refers to adhering to gambling expenditure limits, which is operationalized as a binary outcome, with success defined as actual expenditure being no greater than 10% higher than the planned expenditure limit. Secondary proximal outcomes are strength of intention, goal self-efficacy, and urge self-efficacy. It is hypothesized that action- and coping planning interventions will be associated with higher rates of adherence to gambling expenditure limits compared with the control condition, as well as higher levels of strength of intention, goal self-efficacy, and urge self-efficacy. Should data allow, the secondary aims of this trial are to (1) determine how each of the following influences the intervention effect on adherence to gambling expenditure limits: time-variant (EMA) strength of intention, goal self-efficacy, urge self-efficacy or being in a positive or negative high-risk situation, alcohol or drug consumption, and gambling proximity and time-invariant factors measured before intervention, including age, gender, volitional phase, gambling symptom severity, gambling expenditure, and planning propensity and (2) explore whether the effect of the intervention on adhering to gambling expenditure limits changes over time as the treatment progresses over the course of the 28-day MRT.

## Methods

### Trial Design

A 28-day MRT will be used to facilitate the optimization of *Gambling Habit Hacker*. In this trial, *decision points* comprise notifications that participants will receive via their smartphones to complete a time-based EMA 3 times a day. In each EMA, *tailoring variables*, including strength of intention, goal self-efficacy, urge self-efficacy, and high-risk situations, are used to determine intervention eligibility according to *decision rules* based on EMA cutoff points. At each decision point, participants will be randomly allocated to either the intervention or a control condition (*intervention options*). This MRT will evaluate the JITAI entirely as a *push* intervention.

This MRT will be supplemented with (1) a 6-month within-group follow-up that explores the long-term impact of the app on gambling expenditure (primary distal outcome) and a range of secondary outcomes (gambling frequency, gambling symptom severity, psychological distress, well-being, situational confidence, and planning propensity) as well as the predictors of longer-term treatment outcomes and (2) an evaluation of the acceptability of the JITAI via postintervention surveys, app use and engagement indices, and semistructured interviews.

### Participant Eligibility and Recruitment

Participants will be recruited using a range of strategies, such as web-based advertising, social media, gambling-related websites, and advertisements in public places such as mental health and addiction services, general practices, and universities. Gambling counseling services and gaming venues across Australia and New Zealand may also assist in participant recruitment. The eligibility criteria are (1) current Australian or New Zealand residence, (2) aged ≥18 years, (3) able to install an app on their own smartphone with internet access, (4) willing to have app notifications activated, (5) English fluency, and (6) seeking assistance for their own gambling. The target population is those with less severe gambling problems who want to adhere to their gambling expenditure limits through the promotion of the app. Consistent with a pragmatic design, there is no requirement to meet any problem gambling diagnostic criteria, and participants are able to engage in other help-seeking activities [56]. However, we will measure the severity of gambling problems using the Gambling Symptom Assessment Scale (G-SAS) [57] to determine the gambling symptom severity of gamblers using the app and explore the degree to which gambling symptom severity influences the efficacy of the intervention.

### Participant Time and Reimbursement

The total time required to complete the intervention is approximately 6 hours. This includes registration and follow-up evaluation surveys (1 hour), 3 time-based EMAs per day for 28 days (3 hours), and 2 hours of engagement with the action- and coping planning intervention (5 minutes for the intervention flow at an estimated 24 times over the 28 days). Participants could receive a maximum of Aus $230 (US $160) in e-gift vouchers for participating in this study. Participants will receive Aus $1 (US $0.70) for each completed EMA, a Aus $20 (US $13) bonus if >75% of EMAs are completed (to a maximum of Aus $100 [US $69]), $50 for the posttreatment evaluation, and Aus $50 (US $34) for the 6-month follow-up evaluation. If selected for a semistructured interview (optional participation), interviewees will receive an Aus $30 (US $20) e-gift voucher.

### Onboarding Procedure

Recruitment materials will direct participants to Apple or Android app stores. Once the app is downloaded, potential participants will be directed to review the plain language statement, as well as the terms of use and privacy policy. During this process, potential participants will also be asked to provide informed consent by confirming that they meet the eligibility criteria and are willing to participate in the trial activities (completion of brief surveys, EMAs, and ecological momentary interventions). Participants are advised during the consent process that, during the trial period, the app will present strategies specifically aligned with EMA responses with varying levels of detail. Those who provide consent will create an account for the *Cogniss* platform, including setting a username and password and providing an email address. Following account creation, participants will be required to read a brief app description and information about how the app works. Participants will then be directed to the preintervention survey (including their mobile number, interest in being contacted for further research, and interest in participating in the optional semistructured interview), after which they will be encouraged to complete an EMA.

### Distal and Proximal Outcomes

The *distal outcome* for *Gambling Habit Hacker* is gambling expenditure. The *primary proximal outcome* is goal adherence (operationalized as a binary outcome, with success defined as actual gambling expenditure being no greater than 10% higher than planned gambling expenditure). In exploratory analyses, the impact of altering the flexibility percentage (eg, 20% flexibility) and continuously scaled measures of adherence may also be explored. To specify expenditure goals over the 28-day MRT, participants are required to indicate their intended gambling episodes and associated expenditure during preintervention measurement using a TimeLine Follow-Forward [58]. Actual expenditure will be collated via the event record in each EMA: S*ince the last time you checked in, how much have you spent in $ gambling? Record 0 if you have not gambled*. *Secondary proximal outcomes* are drawn from EMA data, which allows us to examine changes across the course of the intervention. Outcomes include the strength of intention to adhere to gambling goals, goal self-efficacy, and urge self-efficacy, all of which are measured in the subsequent EMA (see the *Tailoring Variables* section for information on the source and description of each variable).

### Decision Points

Participants will take part in a 28-day MRT in which *Gambling Habit Hacker* will administer an EMA protocol that uses time-based sampling that incorporates event-based sampling. Each EMA comprises 18 items that assess influential internal or situational cues and an item that assesses an event record of any gambling expenditure since the previous EMA. The EMA is an active assessment that is undertaken in-app and by self-report, with completion prompted by push notifications delivered to participants at random times during 3 prespecified times during a day: morning (8:30 AM-11:00 AM), afternoon (1:00 PM-3:30 PM), and evening (5:30 PM-8:00 PM). EMA can be auto-launched via notification or through the app. Each EMA takes approximately 2 minutes to complete and is most often measured on a 5-point Likert-type scale with varying response options. In the case where participants are not able to complete the EMA when the push notification is delivered, they will be able to complete the EMA up to two hours after receiving the push notification to accommodate possible unavailability such as driving or working [55,59]. If the EMA is not completed within 2 hours of receiving the push notification, it is no longer accessible and is considered *not completed*.

To enhance EMA compliance at the outset and during the delivery of the intervention, the following contact protocol will be adopted: (1) an automated welcome email when registering, (2) a reminder email to participants who fail to complete onboarding or fail to complete an EMA for >48 hours following onboarding, and (3) a reminder telephone call by qualitatively or clinically trained research fellows who still fail to complete onboarding or an EMA in the subsequent 48-hour period (with a follow-up SMS text message, if no answer or second follow-up email, if they provided no valid phone number). Participants who fail to complete an EMA following this protocol will receive no further contact but will be eligible to receive follow-up evaluations, as long as they complete onboarding, at least one EMA, and have some engagement with the app interventions.

### Tailoring Variables

The eligibility for EMI content is guided by a set of tailoring variables and decision rules [43,45,60-62]. In this study, all 18 EMA items serve as tailoring variables to ensure *Gambling Habit Hacker* delivers the right amount of support at the right time. The choice of tailoring variables is aligned with the HAPA model in terms of strengthening goal intention and addressing internal or situational variables that could weaken intention and the ability to implement and maintain behavioral attention. Gamblers report that motivation is a major barrier to adhering to gambling expenditure limits and that internal and situational factors make it challenging to maintain change [63]. Tailoring variables targeting both the motivation (strength of intention for goal adherence) and volition (being able to implement and maintain actions to limit gambling) phases were selected.

Tailoring variables are presented in Multimedia Appendix 1 [64-66]. The first set of tailoring variables was the strength of intention (*Right now, I intend to meet my goal*) and goal self-efficacy. (*Right now, I am confident that I can stick to my goal*). These variables relate to the proximal outcome of adhering to gambling expenditure limits and were adapted from the study by Schwarzer et al [64]. The second tailoring variable was designed to support urge self-efficacy and focused on the confidence to adhere to gambling limits despite an urge to gamble (*Right now, it would be difficult to turn down a bet*). This variable is measured using a single item from the Gambling Urge Scale [65]. The third set of tailoring variables supports maintenance of self-efficacy (confidence in implementing an action). To do this, the focus is on high-risk situations, which are internal and situational variables that are well-established barriers to adhering to gambling expenditure limits [63]. Participants are asked to indicate whether they were currently experiencing negative reinforcement high-risk situations (*difficulties and conflict or arguments with other people*), positive reinforcement high-risk situations (*thinking that my skill or system could help me to win at gambling*), consuming alcohol or drugs, and gambling proximity (*gambling right now as planned*). Positive and negative reinforcement high-risk situations were drawn from the study by Smith et al [66], with the addition of a single item assessing current physical discomfort (*physically uncomfortable or trouble sleeping*). Alcohol and drug consumption and gambling proximity were single items developed for this study. High-risk situations are assessed on a 5-point Likert scale from 1=*not at all* to 5=*completely*.

### Decision Rules

*Gambling Habit Hacker* determines eligibility for an intervention based on responses to the 18 tailoring variables. A threshold was set for each tailoring variable, which was used to determine whether the person was eligible for an EMI. As shown in Multimedia Appendix 1, the thresholds varied across each tailoring variable. A score of 1 to 3 (strongly disagree to neutral) was the threshold for the strength of intention (to adhere to gambling expenditure limits) and goal self-efficacy. A score of 3 to 5 (neutral to strongly agree) was the threshold for urge self-efficacy. A score of 2 to 5 was the threshold for being in a high-risk situation.

Each strategy group is linked to at least one tailoring variable, whereby 14 strategy groups were identified for strengthening intention to adhere to gambling limits, 12 strategy groups for goal self-efficacy, 16 strategies for urge self-efficacy, and all 25 strategy groups for high-risk situations. As shown in Table 1, each high-risk situation was allocated between 2 and 9 different strategies that could be used to directly address potential risk to adhering to gambling expenditure limits. Some strategies were specific to only 1 or 2 tailoring variables; for example, the strategy *slow down the bets* is only relevant to gambling on an intended gambling day. Other strategy groups, such as *talk to someone*, *seek professional support*, and *improve motivation*, are offered across almost all situations.

If participants reach EMI eligibility on several tailoring variables, a predetermined hierarchy (achieved through researcher consensus) is implemented, in which the app will deliver the EMI relevant for the highest ranked EMA item. The following hierarchy was determined, ranging from the most to least immediate threat to adhering to gambling expenditure limits: gambling proximity (whether the person is currently engaged in planned or unplanned gambling or whether it is a planned gambling day), weakened urge self-efficacy, being in a high-risk situation, weakened strength of intention to adhere to limits, and weakened goal self-efficacy. We consider current gambling to be the most serious high-risk situation for adhering to gambling expenditure limits, whether planned or unplanned.

Importantly, a *provide nothing* option is provided for situations in which the participant ignores the push notification prompting EMA completion or presses the *snooze* function to indicate that they are currently unable to complete the EMA (which suggests that they are not in a state of receptivity).

**Table 1:** Just-In-Time Adaptive Intervention decision rules linking tailoring variable (time-based ecological momentary assessment) with ecological momentary intervention response^a^.

Strength of intention	Goal self-efficacy	Urge self-efficacy	High-risk situations^b^	EMI strategy group activated^c^
✓	✓	✓	Q1, Q4, Q5, Q6, Q7, Q11, and Q14	Build commitment
✓	✓		Q3 and Q8	Build momentum
✓			Q6, Q9, Q10, Q13, and Q14	Control cash in venue
✓	✓	✓	Q1, Q4, Q6, Q8, Q9, Q10, Q12, Q13, and Q14	Control gambling urges
✓	✓	✓	Q1, Q2, Q3, Q4, Q5, Q6, Q7, Q9, Q11, and Q14	Do something else
✓		✓	Q1, Q3, Q4, Q5, Q6, Q11, and Q14	Deal with emotions
		✓	Q2, Q3, Q5, Q7, Q8, Q11, and Q13	Grab a treat
✓		✓	Q3 and Q12	Impose rewards and consequences
	✓		Q8 and Q12	Keep to budget
✓		✓	Q1, Q3, Q6, Q8, Q12, and Q14	Know reasons for change
✓	✓		Q4, Q9, Q12, Q13, Q14, and Q15	Know tricks pokies play
			Q4, Q13, Q14, and Q15	Know when to walk away
	✓	✓	Q1, Q4, Q7, Q8, Q10, Q13, Q14, and Q15	Limit cash
	✓	✓	Q6, Q7, Q8, Q10, Q13, and Q15	Prepokies prep
		✓	Q1, Q4, Q5, Q8, Q9, Q10, Q12, Q13, and Q14	Reduce cash in hand
✓	✓	✓	Q1, Q4, Q5, Q6, Q7, Q9, and Q10	Reduce gambling thoughts
			Q2, Q8, Q10, and Q11	Reduce stress
✓	✓	✓	Q1, Q2, Q4, Q6, Q8, Q13, Q14, and Q15	Strengthen goal
✓			Q3, Q10, Q11, and Q12	Support good health
✓	✓	✓	Q1, Q2, Q3, Q8, Q9, Q10, Q11, Q13, and Q14	Seek professional support
			Q4, Q9, Q13, Q14, and Q15	Self-control in the venue
			Q9 and Q13	Slow down the bets
		✓	Q1, Q4, Q5, Q6, Q7, Q9, Q10, and Q12	Stay away from venues
		✓	Q5, Q6, Q7, and Q11	Swap gambling
✓	✓	✓	Q1, Q2, Q3, Q5, Q6, Q7, and Q14	Talk to someone

^a^On the basis of whether the participant has the current availability to complete the ecological momentary assessment.

^b^Q1: temptations to gamble, such as having money or being reminded of gambling; Q2: difficulties, conflict, or arguments with other people; Q3: unpleasant feelings such as depression, loneliness, or frustration; Q4: wanting to win back money or thinking about winning more; Q5: feeling good and want to gamble today, but today is a no gamble day; Q6: people are encouraging, pressuring, or creating a desire to gamble; Q7: wanting to pass some time; Q8: worry about debt or how you will pay the bills; Q9: thinking that your skill or system could help you to win at gambling; Q10: you are drinking or taking drugs; Q11: physically uncomfortable or having trouble sleeping; Q12: beginning to think that you no longer have a gambling problem; Q13: gambling right now as planned; Q14: gambling right now but not planned; Q15: not gambling right now but a planned gambling day.

^c^Refer to Table 2 for strategies within each group.

### Microrandomization Procedure

**Figure 1:**
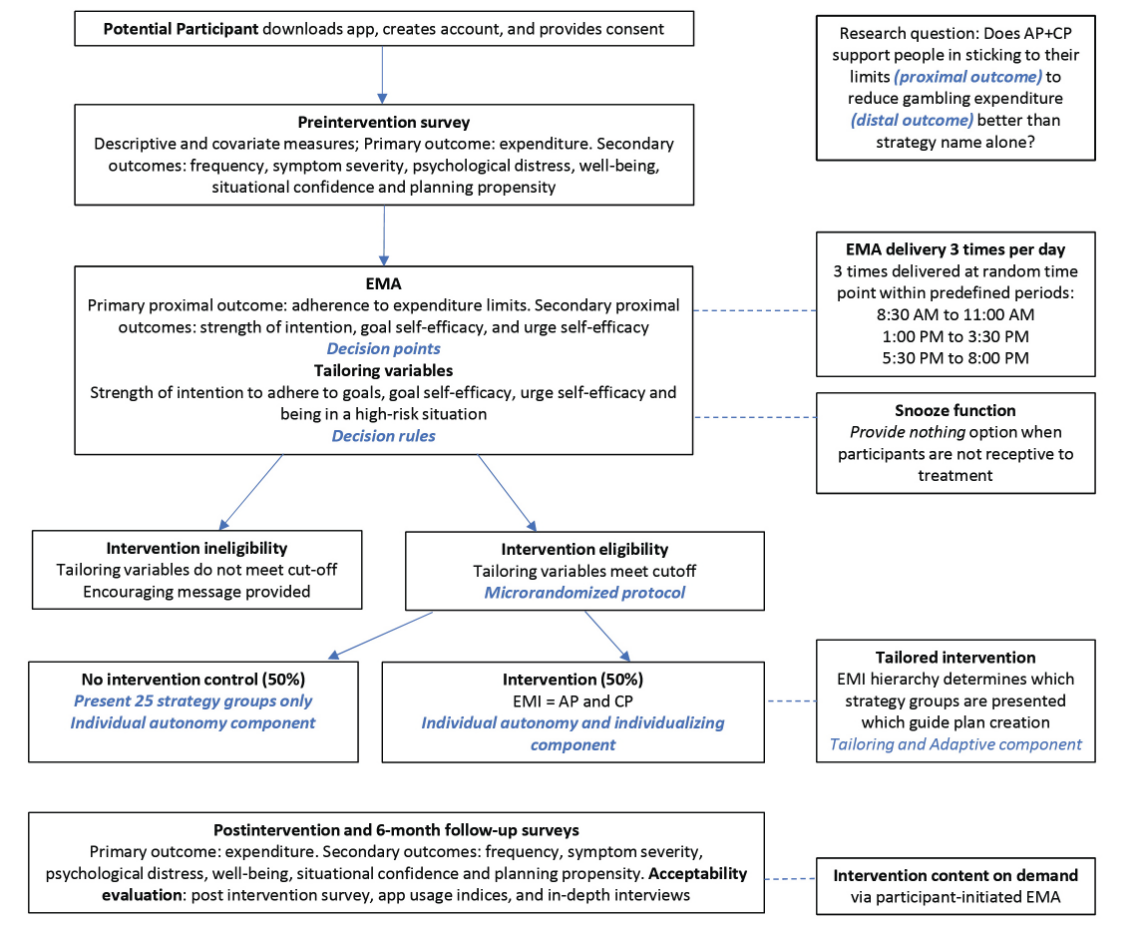
Microrandomized trial design of *Gambling Habit Hacker*. AP: action planning; CP: coping planning; EMA: ecological momentary assessment; EMI: ecological momentary intervention.

Each time a participant completes an EMA, responses will be assessed for intervention eligibility (Figure 1). If a participant is eligible for an EMI at that point in time, they will be randomized to one of the following two conditions: (1) specific strategies with facilitated action- and coping planning or (2) control group (strategy groups—strategy group level names only). Randomization to the 2 conditions occurs in real time within the app algorithm; at the time, the participant becomes eligible for the EMI and occurs on a 50:50 split between control and intervention. The microrandomization procedure used by *Gambling Habit Hacker* uses a fully automated randomization process, whereby a random number generator is embedded in the app. This guarantees that the administration of treatments and assessment of outcomes are fully blinded.

### Intervention Options

#### Overview

The *Gambling Habit Hacker* app is part of a suite of interventions for addictive behaviors that delivers implementation support based on lived experience, along with goal setting, action planning, and coping planning [39,58,67]. In this study, *Gambling Habit Hacker* is delivered as a JITAI that delivers in-the-moment support for adhering to the gambling expenditure limits. The overarching set of principles guiding the *Gambling Habit Hacker* app is the HAPA [32] and self-determination theory [29]. HAPA guided the selection of BCTs: goal setting, action planning, coping planning, and self-monitoring as the mechanism for bridging the gap between intention and behavior. Self-determination theory guided the delivery of BCTs, whereby goal setting and planning were personalized to support individual autonomy and the development of competence [29,35]. In-app communication was nonjudgmental and respectful and aimed to support relatedness through the inclusion of lived experience information and quotes. In accordance with self-determination theory, participants can select any gambling expenditure limit, including reduction or abstinence [29].

#### Intervention Condition—Strategy Groups, Action Planning, and Coping Planning

Gamblers use a wide range of cognitive and behavioral strategies to limit or reduce their gambling behavior [15,17,18,20,23,26,30,68]. To support participants in selecting and implementing the right strategy at the right time, we developed a comprehensive list of strategy options. Each option contains a strategy name such as *social support* and implementation guidance such as *talking to someone about gambling*. Each strategy was categorized into a higher order group, which is referred to as a strategy group (n=25). Each strategy group contains between 4 and 6 strategies, each with detailed implementation guidance (120 strategies in total). For reporting and comparison with the broader literature, strategy groups were organized into 10 higher order BCT categories [14]. As indicated in Table 2, these were avoidance, rewards, substitution activities and social support, and categories of BCTs identified in the gambling literature [15], including maintaining momentum, staying in control while gambling, urge management, financial management, and managing emotions.

**Table 2:** Individual strategies for adhering to gambling expenditure limits organized by behavior change technique (BCT) category and strategy group.

BCT category (n=10) and strategy group (n=25)			Individual strategies (n=120)
**Avoidance**			
	Stay away from venues		Ban yourself; go away now; block online venue; deal with social pressure.
**Financial management**			
	Keep to budget		Calculate cash allowance; get a debit card without cash access; block access to online banking; cancel or destroy credit cards; ensure you cannot draw money from assets; keep wages safe.
	Reduce cash in hand		Pay bills; buy essentials; set up savings account.
	Limit cash		Set a cash limit today; leave cards at home; change personal identification numbers; give cards to a family member; use prepaid cards; reduce automated teller machine limits.
**Maintaining momentum**			
	Build momentum		Know reasons for change; take it a day at a time; take it slow and steady; pause and celebrate.
	Strengthen goal		Keep your plan number 1; slow down the emotional roller coaster; let go of guilt and shame; get inspiration.
	Know reason for change		Identify gambling cons; change pros; know your rock bottom; identify harm; stop chasing losses; accept loss of control.
	Build commitment		Build confidence; build willpower; increase accountability; focus on today.
**Managing emotions**			
	Deal with emotions		Deal with boredom; deal with frustration; deal with pain; deal with anger; deal with loneliness; deal with happiness.
**Rewards**			
	Impose rewards and consequences		Accept a reward; give yourself a reward; create a future reward; set up a penalty system; give yourself a red card.
	Grab a treat		Feel good treat; get creative; connect with someone; achieve something; do something fun.
**Substitution activities**			
	Do something else		Get busy; get moving; connection; get a positive addiction; feel good.
	Swap gambling		Play low-cost games; play no cost pokies; play no cost casino games; get adrenaline in a different way; play other games.
**Social support**			
	Talk to someone		Talk it over; admit or confess; be accountable; get advice; find someone to support your goals.
	Seek professional support		Talk to a gambling counselor; talk to a counselor about mental health; talk to a financial counselor; go to a support group; go to a 12-step group; access online support.
**Staying in control while gambling**			
	Prepokies prep		Set your loss limit; set your time limit; leave cash and cards at home; leave cash and cards in the car; give cash and cards to a friend.
	Control cash in venue		Avoid automated teller machines; do not borrow money; keep winnings.
	Slow down the bets		Change machines often; take breaks; keep the same bet size; take time between spins; avoid chasing losses.
	Know when to walk away		Walk away when limits are reached; walk away when you hit your time limit even if it is fun; walk away when it is no longer fun; walk away from social pressure.
	Increase self-control in venue		Do not get comfortable; view gambling as entertainment; address thoughts of abandoning limits; avoid stimulants.
**Stress management**			
	Reduce stress		Sleep; take a walk; cut stimulants; relax; self-massage.
	Support good health		Eat healthy; cut alcohol; check your mental health; practice mindfulness; exercise.
**Urge management**			
	Control gambling urges		Stay away from a venue; reduce mental tension; walk out of a venue; remove access to cash; deal with payday; do something else; learn to say no.
	Reduce gambling thoughts		Deal with advertising; address thoughts of wining; address thoughts of gambling; deal with permission giving.
	Know tricks pokies play		Let go of lucky charms; remember pokies are based completely on chance; notice losses disguised as wins; remember no skill is needed.

Even though gamblers use a wide range of strategies, studies indicate that up to 80% of gamblers fail to adhere to their gambling limits [25]. Forming specific plans about how, when, and where to act has been shown to increase adherence to gambling limits in gambling venues [39]. The action planning component prompts participants to develop personally tailored action plans that respond to immediate threats to adhering to gambling limits. Participants allocated to the intervention condition received a list of tailored strategy groups (6-16 groups) based on the results of their EMA. Participants can select a strategy group, and the app then provides a list of all relevant individual strategies from which they can select. Upon selection, the app provides a detailed description of methods for implementation (drawn from lived experience research; see the *Consumer Participation* section [15]) and offers strategy-specific prompts for the personalization of the strategy. As an illustration of this process, if the participant were to select *Talk to someone* from the list of strategy groups, they would then be presented with a list of five individual strategies: Talk it over, Admit or confess, Be accountable, Get advice, and Someone to support your goals. If the participant then selected the strategy *Be accountable*, the app will provide the following information: *how to identify someone that will hold you accountable for your plan, different ways to involve others for accountability, how having someone for accountability can make you feel, and what can get in the way of your plans.* The app further prompts the participant to think of specific details, such as the name of the person they will be accountable to; why this person is a good option; and whether they will text, call, chat, or email that person. Once participants are provided with all the information and prompted to consider specific details, they are asked to record their action plan. The app produces the following prompt: *If this strategy sounds good to you, then take action. Write your plan here on how you will* (name of the strategy). *Include in your plan something you can do right now. Be specific about what you will do and how you will do it.* The prompt is followed by an open text field for the participant to detail their personalized, detailed action plan.

Action planning can bridge intention and behavior, but it does not directly address barriers that can get in the way of even the most robust plans. The coping planning component involves the development of a personally tailored coping plan in response to a proximal implementation barrier [69]. Action planning and coping planning are developed simultaneously, based on a series of prompts in the app. Participants are prompted to identify the main barriers to their action plan implementation by selecting 1 of 7 categories: thoughts, emotions, motivation, situation, self-belief, financial, and social. Participants are prompted to describe the selected barrier in an open text field and to detail how that barrier can get in the way of their action plan. The app then prompts participants to identify what they can do right now to overcome the barrier and get back on track with the plan (open text field).

Once the plan is saved, commitment and self-efficacy activities facilitate the strategy’s engagement and throughput. This activity involves focusing on character strength and mental rehearsal [70,71]. Consideration of character strengths is prompted by *Name your strength that can help you stick to your plan and overcome the barrier you have identified. Write down exactly how this strength will be useful* (open text field). To undertake mental rehearsal, the person is prompted to imagine implementing their plans. A prompt for mental imagery is provided as follows:

You have decided what to do, so take a moment to visualize yourself doing it. Close your eyes and imagine yourself doing what you need to do. Imagine it going well. Imagine feeling happy that you take this action. You feel proud of yourself and feel good because you have control. You are ready to do this action right now.

Once completed, the app provides an encouraging message:

Great work in putting together your plan and identifying your strengths. This is really going to help you stick to your gambling goals. We will check in again in a few hours to see how you are going.

#### Control Condition—Strategy Group Names Only

Participants allocated to the control condition will receive a list of all 25 strategy groups (names only), with no specific strategies or implementation guidance provided. Once participants select a strategy group name, such as *Talk to someon*e, the app provides an encouraging message: *It is a great idea to* (insert strategy group name)*. We will get in touch with you soon to check how you are doing*. Providing information at the strategy group level is similar to responsible gambling messaging and reflects a real-world experience of these messages.

### Consumer Participation

People with lived experience of gambling problems are represented in all aspects of *Gambling Habit Hacker*, including identification of intervention content, testing of app functionality, and recommendations for future improvements via postintervention survey items and semistructured interviews. The app includes detailed implementation support, delivering >70,000 words of content. This content was sourced from consumer accounts representing >2000 individuals from various sources, including counseling transcripts [26], in-venue surveys [23], online forums [17], and community-based quantitative and qualitative surveys [15,30]. Within the app, quotes are taken directly from this lived experience research, selected to align with each strategy group.

### Within-Group Evaluation

In addition to MRT, this study will include a within-group follow-up evaluation over a 6-month period to (1) examine within-group change over the 6 months following the end of the MRT and (2) identify predictors of these longer-term treatment outcomes (including usage of the app over the follow-up period). Surveys taking 10 to 15 minutes will be administered before intervention (via the app), as well as after intervention and at the 6-month follow-up (via Qualtrics). Descriptive and covariate measures will include sociodemographic characteristics (age, sex, current residence, primary ethnicity, and personal annual gross income), problem gambling activities (6 types of gambling), and intended gambling behavior as measured by the TimeLine Follow-Forward [58], volitional phase [72], and help seeking [73]. To assess the volitional phase, participants will be presented with 4 statements: I am deciding whether I need to change my gambling (predecisional); I am getting ready to change my gambling (postdecisional); I have already started to change my gambling (actional); and I have successfully changed my gambling and want to maintain this change (postactional). Posttrial and 6-month follow-up participants will be asked to report on their previous help-seeking behavior using 9 items from the Help-Seeking Questionnaire [73]. This includes 5 items related to high-intensity help seeking and 3 items related to low-intensity help seeking. An item related to self-directed help seeking will also be administered to assess engagement with self-exclusion.

The primary outcome for the within-group evaluation will be gambling expenditure (measured using a TimeLine Follow-Back at the preintervention evaluation, the EMA data collected during the intervention period and amalgamated for the posttreatment evaluation, and single items at the 6-month evaluation). Secondary outcomes will include gambling symptom severity measured using the G-SAS [57], gambling frequency with the TimeLine Follow-Back at the preintervention evaluation (EMA data at the posttreatment evaluation and single items at the 6-month evaluation) [74], psychological distress measured using the Kessler 6 Psychological Distress Scale [75], personal well-being measured using the Personal Wellbeing Index [76], the Brief Situational Confidence Questionnaire [77], and planning propensity measured using an adapted action control questionnaire that assesses action planning, coping planning, and action control [78]. A summary of the measures and the measurement time points (before intervention, after intervention, and follow-up) for the within-group evaluation are presented in Multimedia Appendix 2.

To enhance engagement with the posttreatment and 6-month follow-up evaluations, the following protocols will be implemented. An email will be sent to all participants to prompt survey completion, with a second reminder email for those who fail to complete within a week. An advance notice email will also be sent a week before the 6-month surveys are administered. For those who have not completed the survey after a further week, up to two reminder telephone calls will be made by a clinical or qualitatively trained researcher. At each time point, the option to complete the survey over the phone with a trained research fellow will be offered. Participants who do not complete posttreatment evaluation will be contacted at the 6-month evaluation unless they have withdrawn from the study.

During the 6-month evaluation period, *Gambling Habit Hacker* will be available to participants for continued use. The tailored intervention content will be available on demand, meaning that participants can complete EMAs at any time of the day or night. The intervention content will still be tailored, but participants will not receive the 3 times daily prompts for EMA completion. This approach is designed to encourage participants to incorporate action- and coping planning skills in everyday situations and settings when there is a shift in motivation, self-efficacy, or the presence of high-risk situations.

### Acceptability Outcomes

Acceptability is operationalized as a multifaceted construct reflecting the degree to which participants consider *Gambling Habit Hacker* to be appropriate, based on their emotional and cognitive responses to the app [79]. Specifically, *intervention fidelity* will be assessed by the proportional response to EMA notifications, strategy selection, and completion of the written text for EMI action and coping plans. The content of each personalized action and coping plan will also be reviewed and rated: 0=not completed, 1=partially completed (ie, plan is created but is missing key detail on how or what the person will do right now), and 2=completed (ie, plan is created and includes all details as prompted). Subscales of the *Mobile App Rating Scale* [80] will be used to measure the subjective quality (4 items: willingness to recommend the app, future use, willingness to pay, and overall perception of quality) and perceived impact (6 items: awareness, knowledge, attitudes, intention to change, help seeking, and behavior change) of the app. *App use and engagement* will be assessed across the 28-day MRT and 6-month follow-up period by download information, onboarding information, app use information (eg, EMA compliance, intervention eligibility, participant retention, and intervention activities completed), and other evaluation information. A series of *semistructured interviews* will be conducted 28 days after trial with a subsample of 10 participants from the MRT. Participants will be selected based on gender and app use (high or low), with participants prioritizing the state of New South Wales in alignment with the funding source. Participants will be individually interviewed via videoconferencing and asked about their experiences with the *Gambling Habit Hacker* app as well as its perceived helpfulness and areas for improvement.

### Sample Size

A sample size of 200 will be recruited based on a conservative anticipated 40% attrition rate [52], providing a final sample of 120 at 6 months after evaluation. This sample size provides >85% power to detect a small binary outcome intervention effect of relative risk=1.20 (α=.05; availability parameter=0.3; randomization probability=.50; probability of outcome without intervention=.25) [81].

### Statistical Analyses

To assess the research questions, the method of generalized estimating equations will be used, with an appropriate link function for the outcome of interest (eg, logit or identity). Although an exchangeable working correlational structure is intended to be used for the analyses, considerations will be given to alternative correlational structures based on the observed within-person correlation pattern over the course of the study (eg, independent or auto-regressive). For all MRT analyses, the (lagged) outcome of interest (eg, adherence to expenditure limit at Time_t+1_) will be regressed on a variable denoting the treatment received (ie, intervention vs control) at Time_t_, as well as covariates (including unbalanced time). The primary analyses will explore the effect of intervention versus control on the probability of adherence to expenditure limits in the subsequent episode. The identification of the conditions under which the interventions are most beneficial and how the effect of the interventions changes over the course of the MRT will be examined by specifying interaction terms between the intervention variable and interaction variables of interest (eg, strength of intention and time).

The long-term outcomes of the intervention (within-group follow-up evaluation) will be explored, whereby distal outcomes will be assessed using generalized estimating equations by regressing the outcome of interest (eg, gambling expenditure) on a variable denoting time (ie, before intervention, after intervention, and 6-month follow-up) and covariates. The identification of factors predicting longer-term outcomes will also be assessed by regressing the outcome of interest (eg, clinically significant changes in gambling expenditure) with selected preintervention, postintervention, and app usage variables. Where appropriate, missingness will be addressed using multiple imputations with appropriate accounting for the multilevel nature of the data (eg, multilevel multiple imputation).

In addition to the effect sizes for all primary and secondary outcomes, the metrics of individual-level change will be calculated for all primary and secondary outcomes. Changes beyond that attributable to chance or measurement error will be evaluated using Reliable Change Indices [82], and clinically significant changes [83] will subsequently be calculated at postintervention and 6-month evaluation using functional score ranges where possible (G-SAS score of ≤20 and K6 score of ≤13) or at least a 25% improvement in scores [84]. Participants will be identified as *recovered* (final score falls into the functional range and corresponds to a reliable change), *improved* (final score corresponds to a reliable change but falls into the dysfunctional range), *unchanged* (final score does not correspond to a reliable change), or *deteriorated* (final score corresponds to a relative change in the negative direction).

### Ethics Approval

This trial has been approved by the Deakin University Human Research Ethics Committee (2020-304) and prospectively registered with the Australian New Zealand Clinical Trials Registry (ACTRN12622000497707).

## Results

**Figure 2:**
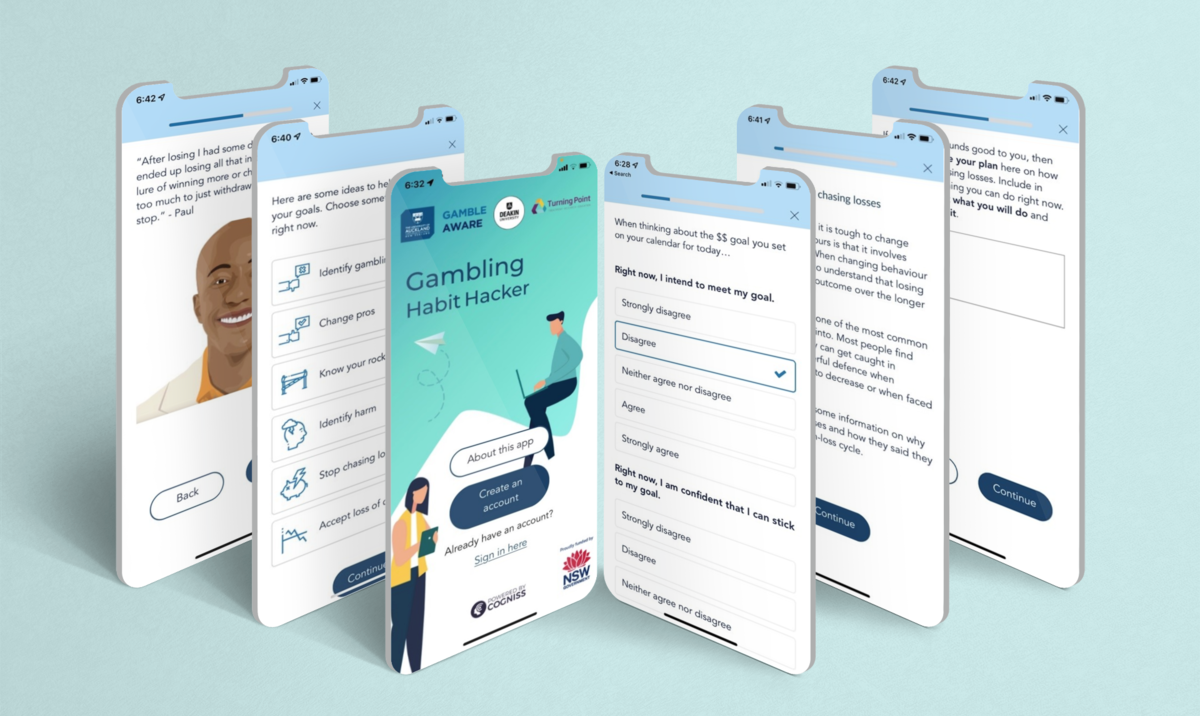
Illustrative screenshots of the *Gambling Habit Hacker* app.

The development and evaluation of *Gambling Habit Hacker* was a collaboration between Deakin University, University of Auckland, Turning Point, and 2and2 (app developers) and funded in June 2019 by the New South Wales Government’s Responsible Gambling Fund. In line with JITAI development recommendations [43], a multidisciplinary team was created to draw on behavior change expertise from clinical and social psychology, implementation science, biostatistics, and research design, in conjunction with smartphone app developers. The development of the treatment content was led by the first author (SNR) and is part of a broader program investigating implementation planning for addictive behaviors. The app is hosted on the Cogniss behavior change platform created by 2and2, a custom technology solution developer. Illustrative screenshots of this JITAI are displayed in Figure 2.

Following an extensive development period, *Gambling Habit Hacker* was subjected to user testing of app functionality between June 2021 and July 2021 with 14 gambling experts. The testers included gambling counselors (n=5), gambling researchers (n=5), and people with lived experience (n=4). Consumers were 3 men and 1 woman, with an average score on the Problem Gambling Severity Index in the problem range (mean 13.0, SD 9.1). Testers used the app on Android and IOS devices for a 3-day period and provided quantitative and qualitative feedback on acceptability, usability, and quality. Quantitative evaluation was performed using the Mobile App Rating Scale [80], which comprises 23 items across 4 subscales measuring perceived engagement, look and feel, functionality, and quality. Experts were reimbursed with an Aus $50 (US $34) e-gift voucher for their time.

Quantitative evaluation indicated that all scores were higher than the minimum acceptability score of 3, which suggests that the app may be helpful [85] (Multimedia Appendix 3). Intervention content was rated highly in terms of strategy relevance to plan creation (>7 out of 10), helpfulness of strategy description (almost 8 out of 10), and quotes on lived experience were perceived as helpful (>7 out of 10). Experts rated planning functionality highly (>7 out of 10) and indicated slightly lower scores for helpfulness of information in coping planning (>6 out of 10). The lowest rated items were TimeLine Follow-Back (calendar to assess past-month gambling behavior) and TimeLine Follow-Forward (calendar to assess planned gambling behavior over the next month), where ease of completion was rated as just over 3 out of 10. Qualitative data were generally positive in terms of comprehensiveness and quality of information, credibility, graphics, and interactivity. The main issues raised were related to functionality in terms of loading times, errors in the notification schedule, and difficulty in entering and saving data in the timeline calendars. The evaluation also indicated that the participant burden needed to be reduced in terms of the length of pretrial measures and the time required to complete the EMA items. Technical issues, including user acceptability concerns, were addressed through extensive redevelopment of the calendars, reducing the number of items in the baseline and EMAs, and correcting the schedule of notifications. A total of 3 testers subsequently used the app again and confirmed that the issues had been satisfactorily resolved.

The user testing phase of the app-administered action planning and coping planning as separate interventions, where coping planning was delivered 30 minutes after action planning and only where participants indicated that they had not implemented their action plan. User testing revealed that this was confusing and was an added burden, which meant most participants did not complete coping plans even if they were assessed as eligible (ie, they had not implemented the action plan). In response to this finding, we merged action- and coping planning as a single intervention delivered concurrently (without a time delay).

The empirical data gathered as part of this trial will be used to optimize the JITAI and to make it more efficient, effective, and scalable. The app is available for download in Australia and New Zealand for Apple (App Store) and Android (Google Play Store) devices. Following its release on the app stores, a total of 7 pilot participants were recruited to check all protocols, and the functionality was operated as intended. Advertising commenced on April 11, 2022, and as of May 26, 2022, a total of 36 participants were recruited for the trial. The trial is expected to conclude in early 2023 with results published mid-2024.

## Discussion

### Overview

Preliminary findings suggest that *Gambling Habit Hacker* is acceptable and feasible for adhering to gambling expenditure limits. To the best of our knowledge, this study is the first to examine the effectiveness of real-time support for implementation planning activities to adhere to gambling expenditure limits. Using an MRT design, this trial will determine if real-time action- and coping planning are more effective than no intervention in adhering to gambling expenditure limits (primary proximal outcome), and using a within-group evaluation, it will determine whether there are reductions in gambling expenditure (distal primary outcome). Consistent with the HAPA model and implementation planning literature, participants are encouraged to specify their gambling intentions (using the TimeLine Follow-Foward) and then use tailored planning to identify opportunities to act. This information will be used to optimize the content of the next version of *Gambling Habit Hacker* in line with recommendations for digital behavior change methods and JITAI development [43,86,87].

Consistent with recommendations [43,87], *Gambling Habit Hacker* is based on well-established behavior change frameworks that delineate intentional and volitional phases, targeting the volitional phase. It is also based on the BCT of goal setting (TimeLine Follow-Forward) with BCTs for specific steps of planning and self-regulation incorporated within the intervention. EMA items are drawn from areas consistently associated with gambling lapses, such as urges and high-risk situations. The EMI content of the active components is designed from the planning literature, specifically examining action- and coping planning. The MRT design allows the evaluation of the intervention components within each subject, providing enhanced statistical power to explore the efficacy of the intervention on real-time variations in behavioral outcomes. All communications within the app are aligned with the self-determination theory, whereby the app offers real-world stories by way of developing relatedness, offers personalized goal setting and planning (autonomy), and provides repeated attempts to practice planning, as well as the identification of barriers that can improve the individual’s chances of adhering to their limits (competence).

Similar to previous studies [36,40], it is proposed that the structure of this intervention could be relevant to any behavior change. We selected adhering to gambling expenditure limits as the proximal goal to be attained, and the strategies presented have come from gamblers’ reports on how to adhere to gambling limits and the associated challenges. EMAs assess the presence of internal and situational cues for unplanned gambling; however, the app design could be adapted to other behaviors where threats to adhering to limits have been identified. The planning framework would remain the same across any health behavior.

Little is known about hourly, daily, or weekly fluctuations in the strength of individuals’ gambling goals or the experience of internal or situational cues associated with unplanned gambling. Although this work will provide such knowledge, there is a risk that the timing and frequency of the EMAs (3 times per day) may be too few on some days and too many on others or that the timing is not aligned with high-risk situations. For example, evening EMAs conclude at 8 PM even though the risk of being in a gambling venue may be outside these hours. Following this, there is a chance that a limited number of EMI-eligible moments will be identified. Although designed for low burden, the repetitive nature of EMA items may lead to low engagement and automatic responses to questions or dropouts. While planning activities are undertaken within the app, implementing the plan is done *in the real world*. A subsequent limitation is that the completion of activity after planning cannot be monitored.

### Dissemination of Findings

The results of this trial will be disseminated to peer-reviewed journals, conference presentations, and stakeholder forums. Plain language findings will be disseminated to participants who indicate an interest in the study findings. Participants can access the findings on project websites [88,89].

### Conclusions

The aim of *Gambling Habit Hacker* is to provide evidence-based, real-time support for individuals wishing to adhere to their gambling expenditure limits. Through the use of the app, individuals’ cognitive burden in identifying, evaluating, selecting, and operationalizing an appropriate option is reduced. The app and associated algorithms present appropriate strategies in line with personalized real-time EMA outcomes.

## Appendix

Multimedia Appendix 1 Tailoring variables collected in the time-based ecological momentary assessment.

Multimedia Appendix 2 Summary of Gambling Habit Hacker time points for components and measures.

Multimedia Appendix 3 User testing: Gambling Habit Hacker expert evaluation scores (n=14).

## Abbreviations

BCT: behavior change technique
EMA: ecological momentary assessment
G-SAS: Gambling Symptom Assessment Scale
HAPA: Health Action Process Approach
JITAI: Just-In-Time Adaptive Intervention
MRT: microrandomized trial

## References

[1] (2013). Diagnostic And Statistical Manual Of Mental Disorders, Fifth Edition.

[2] Neal P, Delfabbro P, O'Neil M (2005). Problem Gambling and Harm: Towards a National Definition.

[3] Calado F, Griffiths MD (2016). Problem gambling worldwide: an update and systematic review of empirical research (2000-2015). J Behav Addict.

[4] (2012). New Zealand 2012 national gambling study: overview and gambling participation. Auckland University of Technology.

[5] Dowling NA, Youssef GJ, Jackson AC, Pennay DW, Francis KL, Pennay A, Lubman DI (2016). National estimates of Australian gambling prevalence: findings from a dual-frame omnibus survey. Addiction.

[6] Gainsbury SM, Russell A, Hing N, Wood R, Lubman DI, Blaszczynski A (2014). The prevalence and determinants of problem gambling in Australia: assessing the impact of interactive gambling and new technologies. Psychol Addict Behav.

[7] Browne M, Greer N, Rawat V, Rockloff M (2017). A population-level metric for gambling-related harm. Int Gambling Stud.

[8] Langham E, Thorne H, Browne M, Donaldson P, Rose J, Rockloff M (2016). Understanding gambling related harm: a proposed definition, conceptual framework, and taxonomy of harms. BMC Public Health.

[9] Dowling NA, Cowlishaw S, Jackson AC, Merkouris SS, Francis KL, Christensen DR (2015). Prevalence of psychiatric co-morbidity in treatment-seeking problem gamblers: a systematic review and meta-analysis. Aust N Z J Psychiatry.

[10] Lorains F, Cowlishaw S, Thomas S (2011). Prevalence of comorbid disorders in problem and pathological gambling: systematic review and meta-analysis of population surveys. Addiction.

[11] Bijker R, Booth N, Merkouris S, Dowling N, Rodda S (2020). Global prevalence of help-seeking for problem gambling: a systematic review and meta-analysis. Addiction.

[12] Cowlishaw S, Merkouris S, Dowling N, Anderson C, Jackson A, Thomas S (2012). Psychological therapies for pathological and problem gambling. Cochrane Database Syst Rev.

[13] Thomas SA, Merkouris SS, Radermacher HL, Dowling NA, Misso ML, Anderson CJ, Jackson AC (2011). Australian guideline for treatment of problem gambling: an abridged outline. Med J Aust.

[14] Michie S, Richardson M, Johnston M, Abraham C, Francis J, Hardeman W, Eccles MP, Cane J, Wood CE (2013). The behavior change technique taxonomy (v1) of 93 hierarchically clustered techniques: building an international consensus for the reporting of behavior change interventions. Ann Behav Med.

[15] Rodda S, Merkouris SS, Abraham C, Hodgins DC, Cowlishaw S, Dowling NA (2018). Therapist-delivered and self-help interventions for gambling problems: a review of contents. J Behav Addict.

[16] Abbott M (2019). Self-directed interventions for gambling disorder. Curr Opin Psychiatry.

[17] Rodda SN, Hing N, Hodgins DC, Cheetham A, Dickins M, Lubman DI (2018). Behaviour change strategies for problem gambling: an analysis of online posts. Int Gambling Stud.

[18] Currie SR, Brunelle N, Dufour M, Flores-Pajot M, Hodgins D, Nadeau L, Young M (2020). Use of self-control strategies for managing gambling habits leads to less harm in regular gamblers. J Gambl Stud.

[19] Matheson FI, Hamilton-Wright S, Kryszajtys DT, Wiese JL, Cadel L, Ziegler C, Hwang SW, Guilcher SJ (2019). The use of self-management strategies for problem gambling: a scoping review. BMC Public Health.

[20] Hing N, Browne M, Russell AM, Rockloff M, Rawat V, Nicoll F, Smith G (2019). Avoiding gambling harm: an evidence-based set of safe gambling practices for consumers. PLoS One.

[21] Currie SR, Hodgins DC, Wang J, el-Guebaly N, Wynne H, Miller NV (2008). Replication of low-risk gambling limits using canadian provincial gambling prevalence data. J Gambl Stud.

[22] Rodda SN, Bagot KL, Cheetham A, Hodgins DC, Hing N, Lubman DI (2018). Types of change strategies for limiting or reducing gambling behaviors and their perceived helpfulness: a factor analysis. Psychol Addict Behav.

[23] Rodda SN, Bagot KL, Manning V, Lubman DI (2019). ‘Only take the money you want to lose’ strategies for sticking to limits in electronic gaming machine venues. Int Gambling Stud.

[24] Rodda SN, Bagot KL, Manning V, Lubman DI (2019). "It was terrible. I didn't set a limit": proximal and distal prevention strategies for reducing the risk of a bust in gambling venues. J Gambl Stud.

[25] Ledgerwood DM, Petry NM (2006). What do we know about relapse in pathological gambling?. Clin Psychol Rev.

[26] Rodda SN, Hing N, Hodgins DC, Cheetham A, Dickins M, Lubman DI (2017). Change strategies and associated implementation challenges: an analysis of online counselling sessions. J Gambl Stud.

[27] Rodda SN, Dowling NA, Knaebe B, Lubman DI (2017). Does SMS improve gambling outcomes over and above access to other e-mental health supports? A feasibility study. Int Gambl Stud.

[28] Sheeran P, Wright CE, Avishai A, Villegas ME, Lindemans JW, Klein WM, Rothman AJ, Miles E, Ntoumanis N (2020). Self-determination theory interventions for health behavior change: meta-analysis and meta-analytic structural equation modeling of randomized controlled trials. J Consult Clin Psychol.

[29] Deci EL, Ryan RM (2008). Self-determination theory: a macrotheory of human motivation, development, and health. Can Psychol/Psychologie canadienne.

[30] Bagot K, Cheetham A, Lubman D, Rodda S (2020). Predictors of strategy engagement for the prevention and reduction of gambling harm: a prospective application of the theory of planned behaviour. Int J Ment Health Addiction.

[31] Armitage C, Conner M (2001). Efficacy of the theory of planned behaviour: a meta-analytic review. Br J Soc Psychol.

[32] Schwarzer R, Luszczynska A (2008). How to overcome health-compromising behaviors. Eur Psychol.

[33] Schwarzer R (2008). Modeling health behavior change: how to predict and modify the adoption and maintenance of health behaviors. Applied Psychol.

[34] Gollwitzer PM (1999). Implementation intentions: strong effects of simple plans. Am Psychol.

[35] Sheeran P (2002). Intention—behavior relations: a conceptual and empirical review. Eur Rev Soc Psychol.

[36] Sniehotta FF, Schwarzer R, Scholz U, Schüz B (2005). Action planning and coping planning for long-term lifestyle change: theory and assessment. Eur J Soc Psychol.

[37] McWilliams L, Bellhouse S, Yorke J, Lloyd K, Armitage CJ (2019). Beyond "planning": a meta-analysis of implementation intentions to support smoking cessation. Health Psychol.

[38] Malaguti A, Ciocanel O, Sani F, Dillon JF, Eriksen A, Power K (2020). Effectiveness of the use of implementation intentions on reduction of substance use: a meta-analysis. Drug Alcohol Depend.

[39] Rodda SN, Bagot KL, Manning V, Lubman DI (2020). An exploratory RCT to support gamblers' intentions to stick to monetary limits: a brief intervention using action and coping planning. J Gambl Stud.

[40] Sniehotta FF, Scholz U, Schwarzer R (2005). Bridging the intention–behaviour gap: planning, self-efficacy, and action control in the adoption and maintenance of physical exercise. Psychol Health.

[41] Verhoeven AA, Adriaanse MA, de Ridder DT, de Vet E, Fennis BM (2013). Less is more: the effect of multiple implementation intentions targeting unhealthy snacking habits. Eur J Soc Psychol.

[42] Vinkers CD, Adriaanse MA, Kroese FM, de Ridder DT (2015). Better sorry than safe: making a plan B reduces effectiveness of implementation intentions in healthy eating goals. Psychol Health.

[43] Nahum-Shani I, Smith S, Spring B, Collins L, Witkiewitz K, Tewari A, Murphy SA (2018). Just-in-time adaptive interventions (JITAIs) in mobile health: key components and design principles for ongoing health behavior support. Ann Behav Med.

[44] Shiffman S (2009). Ecological momentary assessment (EMA) in studies of substance use. Psychol Assess.

[45] Wang L, Miller LC (2020). Just-in-the-moment adaptive interventions (JITAI): a meta-analytical review. Health Commun.

[46] Gustafson DH, McTavish FM, Chih M, Atwood AK, Johnson RA, Boyle MG, Levy MS, Driscoll H, Chisholm SM, Dillenburg L, Isham A, Shah D (2014). A smartphone application to support recovery from alcoholism: a randomized clinical trial. JAMA Psychiatry.

[47] Carpenter SM, Menictas M, Nahum-Shani I, Wetter DW, Murphy SA (2020). Developments in mobile health just-in-time adaptive interventions for addiction science. Curr Addict Rep.

[48] Humphrey G, Newcombe D, Whittaker R, Parag V, Bullen C (2019). SPGeTTI: a smartphone-based problem gambling evaluation and technology testing initiative final report. The University of Auckland.

[49] Coral R, Esposito F, Weinstock J (2020). Don't go there: a zero-permission geofencing app to alleviate gambling disorders. Proceedings of the 2020 IEEE 17th Annual Consumer Communications & Networking Conference (CCNC).

[50] Merkouris SS, Hawker CO, Rodda SN, Youssef GJ, Dowling NA (2020). Gamblingless: curb your urge: development and usability testing of a smartphone-delivered ecological momentary intervention for problem gambling. Int Gambl Stud.

[51] Khazaal Y, Monney G, Richter F, Achab S (2017). « Jeu-contrôle », rationnel d’une application de soutien aux limites de jeux. J de Thérapie Comportementale et Cognitive.

[52] Hawker CO, Merkouris SS, Youssef GJ, Dowling NA (2021). A smartphone-delivered ecological momentary intervention for problem gambling (gamblingless: curb your urge): single-arm acceptability and feasibility trial. J Med Internet Res.

[53] Wright C, Dietze PM, Agius PA, Kuntsche E, Livingston M, Black OC, Room R, Hellard M, Lim MS (2018). Mobile phone-based ecological momentary intervention to reduce young adults' alcohol use in the event: a three-armed randomized controlled trial. JMIR Mhealth Uhealth.

[54] O'Donnell R, Richardson B, Fuller-Tyszkiewicz M, Staiger PK (2019). Delivering personalized protective behavioral drinking strategies via a smartphone intervention: a pilot study. Int J Behav Med.

[55] Klasnja P, Hekler EB, Shiffman S, Boruvka A, Almirall D, Tewari A, Murphy SA (2015). Microrandomized trials: an experimental design for developing just-in-time adaptive interventions. Health Psychol.

[56] Treweek S, Zwarenstein M (2009). Making trials matter: pragmatic and explanatory trials and the problem of applicability. Trials.

[57] Kim SW, Grant JE, Potenza MN, Blanco C, Hollander E (2009). The Gambling Symptom Assessment Scale (G-SAS): a reliability and validity study. Psychiatry Res.

[58] Park JJ, Booth N, Bagot KL, Rodda SN (2020). A brief internet-delivered intervention for the reduction of gaming-related harm: a feasibility study. Comput Human Behav Report.

[59] Goldstein SP, Evans BC, Flack D, Juarascio A, Manasse S, Zhang F, Forman EM (2017). Return of the JITAI: applying a just-in-time adaptive intervention framework to the development of m-health solutions for addictive behaviors. Int J Behav Med.

[60] Heron K, Smyth J (2010). Ecological momentary interventions: incorporating mobile technology into psychosocial and health behaviour treatments. Br J Health Psychol.

[61] Nahum-Shani I, Smith S, Spring BJ, Collins LM, Witkiewitz K, Tewari A, Murphy SA Just-in-time adaptive interventions (JITAIs) in mobile health: key components and design principles for ongoing health behavior support. The Methodology Center, Penn State.

[62] Nahum-Shani I, Hekler EB, Spruijt-Metz D (2015). Building health behavior models to guide the development of just-in-time adaptive interventions: a pragmatic framework. Health Psychol.

[63] Marlatt G, Donovan D (2005). Relapse Prevention: Maintenance Strategies in the Treatment of Addictive Behaviors, 2nd Ed.

[64] Schwarzer R, Lippke S, Luszczynska A (2011). Mechanisms of health behavior change in persons with chronic illness or disability: the Health Action Process Approach (HAPA). Rehabil Psychol.

[65] Raylu N, Oei TP (2004). The gambling urge scale: development, confirmatory factor validation, and psychometric properties. Psychol Addictive Behav.

[66] Smith C, Stewart SH, O'Connor RM, Collins P, Katz J (2011). Development and psychometric evaluation of a 10-item short form inventory of gambling situations. J Gambl Stud.

[67] Brittain M, Consedine N, Bagot KL, Booth N, Rodda SN (2021). Sugar Habit Hacker: initial evidence that a planning intervention reduces sugar intake. J Behav Addict.

[68] Bischof A, Bischof G, Meyer C, John U, Hodgins DC, Rumpf H (2019). Untreated pathological gamblers: who recovers and who does not?. Int Gambl Stud.

[69] Armitage CJ (2009). Effectiveness of experimenter-provided and self-generated implementation intentions to reduce alcohol consumption in a sample of the general population: a randomized exploratory trial. Health Psychol.

[70] Knäuper B, Roseman M, Johnson PJ, Krantz LH (2009). Using mental imagery to enhance the effectiveness of implementation intentions. Curr Psychol.

[71] Hamilton K, Keech JJ, Peden AE, Hagger MS (2019). Protocol for developing a mental imagery intervention: a randomised controlled trial testing a novel implementation imagery e-health intervention to change driver behaviour during floods. BMJ Open.

[72] Heckhausen H, Gollwitzer PM (1987). Thought contents and cognitive functioning in motivational versus volitional states of mind. Motiv Emot.

[73] Rodda SN, Dowling NA, Lubman DI (2018). Gamblers seeking online help are active help-seekers: time to support autonomy and competence. Addict Behav.

[74] Weinstock J, Whelan JP, Meyers AW (2004). Behavioral assessment of gambling: an application of the timeline followback method. Psychol Assess.

[75] Kessler RC, Andrews G, Colpe LJ, Hiripi E, Mroczek DK, Normand SL, Walters EE, Zaslavsky AM (2002). Short screening scales to monitor population prevalences and trends in non-specific psychological distress. Psychol Med.

[76] International Wellbeing Group (2013). Personal Wellbeing Index: 5th Edition.

[77] Breslin F, Sobell LC, Sobell MB, Agrawal S (2000). A comparison of a brief and long version of the situational confidence questionnaire. Behav Res Ther.

[78] Reyes Fernández B, Knoll N, Hamilton K, Schwarzer R (2016). Social-cognitive antecedents of hand washing: action control bridges the planning-behaviour gap. Psychol Health.

[79] Sekhon M, Cartwright M, Francis JJ (2017). Acceptability of healthcare interventions: an overview of reviews and development of a theoretical framework. BMC Health Serv Res.

[80] Stoyanov SR, Hides L, Kavanagh DJ, Zelenko O, Tjondronegoro D, Mani M (2015). Mobile app rating scale: a new tool for assessing the quality of health mobile apps. JMIR Mhealth Uhealth.

[81] Qian T, Walton AE, Collins LM, Klasnja P, Lanza ST, Nahum-Shani I, Rabbi M, Russell MA, Walton MA, Yoo H, Murphy SA (2022). The microrandomized trial for developing digital interventions: experimental design and data analysis considerations. Psychol Methods.

[82] Christensen L, Mendoza JL (1986). A method of assessing change in a single subject: an alteration of the RC index. Behav Ther.

[83] Jacobson NS, Truax P (1991). Clinical significance: a statistical approach to defining meaningful change in psychotherapy research. J Consulting Clin Psychol.

[84] Maust D, Cristancho M, Gray L, Rushing S, Tjoa C, Thase M (2012). Psychiatric rating scales. Handbook of Clinical Neurology.

[85] Masterson Creber RM, Maurer MS, Reading M, Hiraldo G, Hickey KT, Iribarren S (2016). Review and analysis of existing mobile phone apps to support heart failure symptom monitoring and self-care management using the Mobile Application Rating Scale (MARS). JMIR Mhealth Uhealth.

[86] Kok G, Gottlieb NH, Peters GY, Mullen PD, Parcel GS, Ruiter RA, Fernández ME, Markham C, Bartholomew LK (2016). A taxonomy of behaviour change methods: an Intervention Mapping approach. Health Psychol Rev.

[87] Michie S, Yardley L, West R, Patrick K, Greaves F (2017). Developing and evaluating digital interventions to promote behavior change in health and health care: recommendations resulting from an international workshop. J Med Internet Res.

[88] Change strategies project. Change Strategies.

[89] GamblingLess. Deakin University.

