# Supplementary material for: Smartphone App Delivery of a Just-In-Time Adaptive Intervention for Adult Gamblers (Gambling Habit Hacker): Protocol for a Microrandomized Trial
Source: JMIR Res Protoc. 2022 Jul 26;11(7):e38919. doi: 10.2196/38919 (PMC9364163; doi:10.2196/38919)
Supplement: Multimedia Appendix 1 [file resprot_v11i7e38919_app1.docx]

Multimedia Appendix 1: Tailoring variables collected in the Time-Based Ecological Momentary Assessment (EMA)

| **Construct** | **Assessment item** | **Response options** | **EMI eligibility** |
| --- | --- | --- | --- |
| Strength of Intention [64] | When thinking about the $$ goal you set on your calendar for today… Right now, I intend to meet my goal. | (1) Strongly disagree; (2) Disagree; (3) Neither agree nor disagree; (4) Agree; (5) Strongly agree | EMI eligible if scores 1-3 |
| Goal self-efficacy [64] | When thinking about the $$ goal you set on your calendar for today… Right now, I am confident that I can stick to my goal. | (1) Strongly disagree; (2) Disagree; (3) Neither agree nor disagree; (4) Agree; (5) Strongly agree | EMI eligible if scores 1-3 |
| Urge self-efficacy [65] | Right now, it would be difficult to turn down a bet. | (1) Strongly disagree; (2) Disagree; (3) Neither agree nor disagree; (4) Agree; (5) Strongly agree | EMI eligible if scores 3-5 |
| High risk situation [66] | Are you experiencing any of these at the moment? (1) Temptations to gamble such as having money or reminded of gambling (2) Difficulties, conflict or arguments with other people (3) Unpleasant feelings such as depression, loneliness or frustration (4) Wanting to win back money or thinking about winning more (5) Feeling good and want to gamble today but today is a no gamble day (6) People are encouraging, pressuring or creating a desire to gamble (7) Wanting to pass some time (8) Worrying about debt or how to pay the bills (9) Thinking that my skill or system could help me to win at gambling (10) Drinking, taking drugs (11) Physically uncomfortable, trouble sleeping (12) Starting to think you no longer have a gambling problem; (13) gambling right now as planned; (14) gambling right now but not planned (15) not gambling right now but a planned gambling day. | Response options: (1) Not at all (2) A little bit (3) Somewhat (4) Very much so (5) Completely  Negative reinforcement items: 1, 2, 3, 6, 8, 12  Positive reinforcement items: 4, 5, 7, 9, 11.  Alcohol consumption: 10  Gambling proximity: 13, 14, 15 | EMI eligible if scores 2-5 |
