# Supplementary material for: Smartphone App Delivery of a Just-In-Time Adaptive Intervention for Adult Gamblers (Gambling Habit Hacker): Protocol for a Microrandomized Trial
Source: JMIR Res Protoc. 2022 Jul 26;11(7):e38919. doi: 10.2196/38919 (PMC9364163; doi:10.2196/38919)
Supplement: Multimedia Appendix 2 [file resprot_v11i7e38919_app2.docx]

Multimedia Appendix 2: Summary of *Gambling Habit Hacker* time points for components and measures

| **Component / Construct** | **Description / Measurement** | **Pre-intervention** | **Post-intervention follow-up** | **6-month follow-up** |
| --- | --- | --- | --- | --- |
| **Descriptive and covariate measures** | |  |  |  |
| Sociodemographic characteristics | Single items for age, sex, country of residence, ethnicity and annual personal gross income. | X |  |  |
| Problem gambling form | Participants indicate whether they have an issue with any of these gambling types with yes/no: Number games like lotteries, keno, powerball or bingo; pokies or electronic gaming machines; informal private betting for money like playing cards at home; table games like blackjack, roulette, and poker; horses or harness racing or greyhounds; and sports or events. | X |  |  |
| Intended gambling behaviour (expenditure and frequency) | Timeline Follow-Forward is a calendar based system to project future gambling expenditure and frequency [58]. At post-intervention, intended gambling behaviour will be measured using two single items: (1) Over the next six months, how many days per month do you intend to gamble? (intended gambling frequency) and (2) Over the next six months, how much money per month do you intend to spend on gambling? (intended gambling expenditure). | X |  |  |
| Volitional phase | Participants select from: I am deciding whether I need to change my gambling (Pre-decisional); I am getting ready to change my gambling (Post-decisional); I have already started to change my gambling (Actional); and I have successfully changed my gambling and want to maintain this change (Post-actional) [72]. | X | X | X |
| Help-seeking | 9-items from the Help-Seeking Questionnaire [73] which assess engagement with in-person gambling counselling, gambling helpline, online counselling, email counselling, peer support group, generalist counselling, financial counselling, residential facility and self-exclusion. |  | X | X |
| **Primary outcome measure** | |  |  |  |
| Gambling expenditure | Gambling expenditure will be assessed using a 28-day TLFB [74] at pre-intervention; using the 28-day EMA data at post-intervention; and using the single item *“Over the past six months, how much money per month have you spent on gambling?”* | X | X | X |
| **Secondary outcome measures** | |  |  |  |
| Gambling symptom severity | Gambling symptom severity which will be measured with the Gambling Symptom Assessment Scale [57]. The 12-item G-SAS assesses past week strength and frequency of urges and gambling behaviours. Items are scored on a 5-point scale, with varying response options. Scores on the G-SAS range from 0 to 48, with higher scores suggestive of greater gambling symptom severity. These scores can be categorised as mild (8-20), moderate (21-30), severe (31-40) or extreme (41-48). | X | X | X |
| Gambling frequency | Timeline Follow-Back is a calendar based system that prompts participants to enter their gambling frequency in a calendar based format [74]. Frequency is calculated based on the number of gambling events entered into the calendar. | X | X | X |
| Psychological distress | Psychological distress which will be measured with the six item Kessler Psychological Distress Scale (K6) [75]. The K6 uses a past month timeframe to assess general psychological distress. Consistent with Australian scoring a five-point frequency scale is used (1=all of the time, 5=none of the time), with scores summed resulting in a range of 6 to 30. | X | X | X |
| Personal Wellbeing | Wellbeing which will be measured with the Personal Wellbeing Index [76]. This is a 7-item scale assessing the domains of: overall satisfaction, standard of living, health, achieving in life, personal relationships, safety, community-connectedness, and future security. The administration is preceded with an overall question of “Thinking about your own life and personal circumstances, how satisfied are you with your life as a whole?” (subjective wellbeing). Responses are on an 11 point satisfaction scale (0=no satisfaction at all to 10=completely satisfied). Scoring is an average of all domain scores (range of 0 to 11). | X | X | X |
| Situational confidence | Situational confidence which will be measured using the 8-item Brief Situational Confidence Questionnaire. This measure presents high-risk situations and assesses confidence in the ability to resist the behaviour [77]. This Brief Situational Confidence Questionnaire was adapted for gambling by adding three additional items that have previously been employed in gambling populations: confidence in resisting the urge to gamble when faced with situations involving financial pressures, filling time, and alcohol or drugs. Responses to items relating to confidence in resisting gambling across high-risk gambling situations are on a visual analogue scale, ranging from 0 (not at all confident) to 100 (totally confident). | X | X | X |
| Planning propensity | Planning propensity measures for Action Planning (3 items), Coping planning (3 items) and Action Control (4 items) were adapted for gambling from previous intention implementation research [78]. Action planning was assessed following the stem of “During the last four weeks, I have made a specific and detailed plan for…” and the items “how much”, “when”, and “where to gamble”. Coping planning was assessed after the stem “I knew what to do if …” and the items “something interfered with sticking to my $ limits”, “I gambled more than my $ limit”, and “I was in situations where I usually gamble a lot”*.* Action control was assessed following the stem: “During the last four weeks…” and the items: “I watched consistently how often and how much I gambled”, “I often thought about my intention to stick to my gambling limits”, “I have really tried to stick to my $ limit”, and “I tried my best to limit my gambling”. Responses will be recorded on a 7-point agreement scale, ranging from 1 (disagree strongly) to 7 (agree strongly). | X | X | X |
