# Supplementary material for: Smartphone App Delivery of a Just-In-Time Adaptive Intervention for Adult Gamblers (Gambling Habit Hacker): Protocol for a Microrandomized Trial
Source: JMIR Res Protoc. 2022 Jul 26;11(7):e38919. doi: 10.2196/38919 (PMC9364163; doi:10.2196/38919)
Supplement: Multimedia Appendix 3 [file resprot_v11i7e38919_app3.docx]

Multimedia Appendix 3: User testing *Gambling Habit Hacker* expert evaluation scores (n=14)

| Mobile App Rating Scale subscale scores | Mean (SD) |
| --- | --- |
| Overall App Quality *(scored out of 5)* | 3.43 (1.02) |
| Perceived Impact *(scored out of 5)* |  |
| Awareness | 4.0 (1.11) |
| Knowledge | 4.0 (1.11) |
| Attitudes | 3.86 (1.10) |
| Intention to change | 3.86 (1.17) |
| Help-seeking | 4.07 (1.07) |
| Behaviour Change | 3.64 (1.34) |
| Intervention content *(scored out of 10)* |  |
| TLFB calendar |  |
| Helpfulness of instruction | 4.50 (3.59) |
| Ease to complete | 3.43 (3.30) |
| TLFF calendar |  |
| Helpfulness of instruction | 5.07 (3.43) |
| Ease to complete | 3.50 (3.46) |
| Strategy implementation information |  |
| Helpfulness of description | 7.71 (1.64) |
| Relevance of strategies | 7.50 (2.50) |
| Helpfulness for plan completion | 7.43 (2.47) |
| Action and coping planning |  |
| Ease of plan completion | 7.50 (2.47) |
| Helpfulness of information on coping plans | 6.71 (4.80) |
| Helpfulness of lived experience quotes | 7.50 (2.41) |
| Check-ins *(scored out of 10)* |  |
| Clarity of app description | 7.43 (2.17) |
| Understanding of what was required | 7.07 (2.40) |
| Ease of trial sign up | 6.36 (2.68) |
| EMA relevance | 7.29 (2.33) |
| EMA ease of completion | 7.43 (2.34) |

TLFB=TimeLine FollowBack, TLFFF=TimeLine FollowForward, EMA=Ecological Momentary assessment
